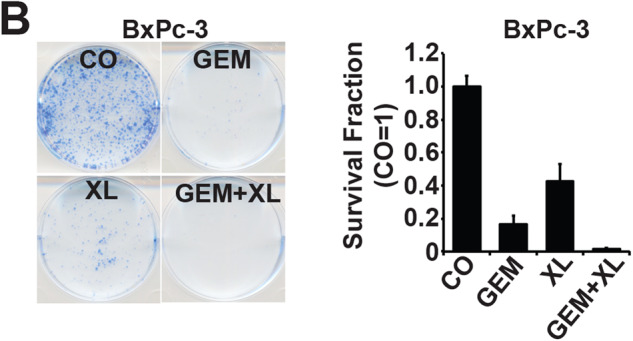# Correction: The novel c-Met inhibitor cabozantinib overcomes gemcitabine resistance and stem cell signaling in pancreatic cancer

**DOI:** 10.1038/s41419-023-06385-9

**Published:** 2024-01-29

**Authors:** C. Hage, V. Rausch, N. Giese, T. Giese, F. Schönsiegel, S. Labsch, C. Nwaeburu, J. Mattern, J. Gladkich, I. Herr

**Affiliations:** 1 Section Surgical Research, Molecular OncoSurgery, University Clinic of Heidelberg, Heidelberg, Germany; 2 Department of General Surgery, Institute for Immunology, University of Heidelberg, Heidelberg, Germany; 3 Department of Molecular Immunodiagnostics, Institute for Immunology, University of Heidelberg, Heidelberg, Germany

Correction to: *Cell Death and Disease* 10.1038/cddis.2013.158, published online 09 May 2013

An image was mistakenly switched in Figure 5B during graphical figure assembly. I would like to emphasize that this image mix-up does not affect the quantitative analysis and the statement of our manuscript in any way. The authors would like to apologize for the confusion.